# Direct fiber vector eigenmode multiplexing transmission seeded by integrated optical vortex emitters

**DOI:** 10.1038/lsa.2017.148

**Published:** 2018-03-09

**Authors:** Jun Liu, Shi-Mao Li, Long Zhu, An-Dong Wang, Shi Chen, Charalambos Klitis, Cheng Du, Qi Mo, Marc Sorel, Si-Yuan Yu, Xin-Lun Cai, Jian Wang

**Affiliations:** 1 Wuhan National Laboratory for Optoelectronics, School of Optical and Electronic Information, Huazhong University of Science and Technology, Wuhan 430074, China; 2 State Key Laboratory of Optoelectronic Materials and Technologies and School of Physics and Engineering, Sun Yatsen University, Guangzhou 510275, China; 3 School of Engineering, University of Glasgow, Rankine Building, Oakfield Avenue, Glasgow G12 8LT, UK; 4 Fiberhome Telecommunication Technologies Co. Ltd, Wuhan 430074, China

**Keywords:** fiber-optic communications, fiber vector eigenmode, multiplexing, optical vortex, photonic integrated devices

## Abstract

Spatial modes have received substantial attention over the last decades and are used in optical communication applications. In fiber-optic communications, the employed linearly polarized modes and phase vortex modes carrying orbital angular momentum can be synthesized by fiber vector eigenmodes. To improve the transmission capacity and miniaturize the communication system, straightforward fiber vector eigenmode multiplexing and generation of fiber-eigenmode-like polarization vortices (vector vortex modes) using photonic integrated devices are of substantial interest. Here, we propose and demonstrate direct fiber vector eigenmode multiplexing transmission seeded by integrated optical vortex emitters. By exploiting vector vortex modes (radially and azimuthally polarized beams) generated from silicon microring resonators etched with angular gratings, we report data-carrying fiber vector eigenmode multiplexing transmission through a 2-km large-core fiber, showing low-level mode crosstalk and favorable link performance. These demonstrations may open up added capacity scaling opportunities by directly accessing multiple vector eigenmodes in the fiber and provide compact solutions to replace bulky diffractive optical elements for generating various optical vector beams.

## Introduction

Optical vortices have attracted increasing interest since the early description as the basic properties of ‘dislocations in wave trains’^1^. The following research into optical vortices became the core of what is now known as ‘singular optics’^2^. Optical vortices feature doughnut intensity profiles possessing phase or polarization singularities, commonly referred to as phase vortices or polarization vortices. An optical phase vortex has a helical phase front that carries an orbital angular momentum (OAM)^3^. An optical polarization vortex, which is a type of vector beam, has spatially variant polarizations and resultant undetermined polarization and null intensity at the beam center^4^. These characteristics are different from those of traditional plane waves with spatially uniform phases and polarizations. In the past decades, optical vortices have been extensively studied in a variety of fields such as optical manipulation, trapping, tweezers, microscopy, imaging, material processing, astronomy, and quantum processing^3, 4, 5, 6, 7, 8, 9, 10, 11, 12^. Beyond these diverse developments, optical vortices, as one type of spatial mode accessing the spatial domain of light waves, have recently been exploited in free-space and fiber optical communications^13, 14, 15, 16, 17, 18, 19, 20, 21, 22, 23, 24, 25, 26^, either by encoding M-ary information as high-dimensional vortex states for modulation^13, 16, 17, 23, 25, 26^ or by employing optical vortices as information carriers for multiplexing^14, 15, 18, 19, 20, 21, 22, 24, 25, 26^. The latter approach using OAM mode multiplexing for improved transmission capacity is analogous to the well-known space-division multiplexing in fiber optical communications based on linearly polarized (LP) modes with homogeneous polarizations^27^. Remarkably, OAM modes (optical phase vortices) and LP modes can be synthesized using different linear combinations of fiber vector eigenmodes. Actually, somehow, the fiber vector eigenmodes manifest optical polarization vortices. For example, the transverse magnetic (TM_01_) mode in a fiber with no magnetic field in the direction of propagation is a radially polarized mode, while the transverse electric (TE_01_) mode in a fiber with no electric field in the direction of propagation is an azimuthally polarized mode. Both the radially polarized mode and azimuthally polarized mode are optical polarization vortices with polarization singularity at the beam center. Accordingly, it will be interesting and worthwhile to exploit optical polarization vortices and directly use fiber vector eigenmode multiplexing for data transmission.

Various techniques for the generation of optical vortices have been demonstrated, including laser cavities, mode converters, spatial light modulators, spiral phase plates, q-plates, fibers, and metamaterials^4, 5, 19, 24, 25, 26^. Perhaps the most convenient way to generate optical vortices is to use the commercially available spatial light modulators^28^, which, however, are relatively bulky and expensive despite the impressive performance. Recently, integrated OAM-carrying optical vortex emitters have been reported^29, 30^. Photonic integration is clearly the trend and the key enabler toward compact, reliable and low-cost optical devices that are required in fiber-optic communications. In this scenario, a laudable goal is to employ photonic integrated devices to generate optical polarization vortices (vector vortex modes) for the excitation and multiplexing transmission of fiber vector eigenmodes.

In this article, we propose and demonstrate the multiplexing and kilometer-scale data transmission of two fiber vector eigenmodes (TM_01_ and TE_01_) generated via integrated optical vortex emitters. Two fiber vector eigenmodes, each carrying a 10-Gbit s^−1^ quadrature phase-shift keying (QPSK) or a 20-Gbit s^−1^ 16-ary quadrature amplitude modulation (16-QAM) signal, are generated using silicon microring resonators etched with angular gratings and then multiplexed for transmission though a 2-km large-core fiber (LCF) link. The measured crosstalk between two fiber vector eigenmodes is approximately −16 dB. The obtained results show favorable performance of data-carrying fiber vector eigenmode excitation and multiplexing transmission.

## Materials and methods

### Concept and principle of direct fiber vector eigenmode multiplexing transmission

**Figure 1:**
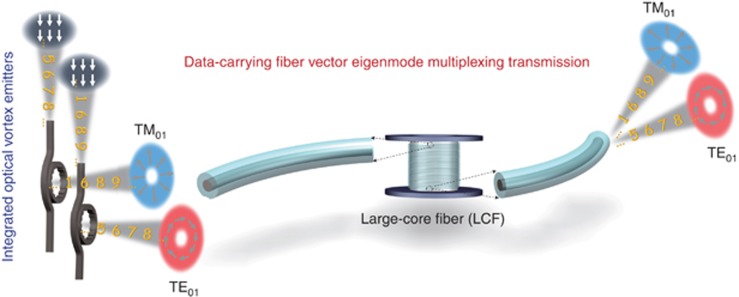
Conceptual illustration of data-carrying fiber vector eigenmode multiplexing transmission seeded by integrated optical vortex emitters. Two data-carrying fiber vector eigenmodes with polarization vortices, that is, radially polarized mode (TM_01_) and azimuthally polarized mode (TE_01_) generated using silicon microring resonators with the inner sidewall etched as angular gratings, are multiplexed and transmitted through a large-core fiber (LCF) for increased transmission capacity and separated after transmission with low-level crosstalk.

The concept and principle of direct fiber vector eigenmode multiplexing transmission seeded by integrated optical vortex emitters are illustrated in Figure 1. High-order fiber vector eigenmodes TM_01_ and TE_01_, which are supported in a LCF, are considered (Supplementary Information). Instead of using commercially available bulky spatial light modulators, two compact integrated optical vortex emitters are employed to generate TM_01_ and TE_01_ modes feeding the LCF for fiber vector eigenmode multiplexing transmission. When each fiber vector eigenmode carries an independent data information channel, the multiplexing of two orthogonal fiber vector eigenmodes can double the transmission capacity through the fiber link, at the output of which the two fiber vector eigenmodes are separable from each other with low-level crosstalk.

### Integrated optical vortex emitters

The designed and fabricated integrated optical vortex emitters are silicon microring resonators with the inner sidewall etched as angular gratings that can extract the confined whispering gallery modes (WGMs) in the microring resonator into radiated optical vortices^29^. The so-called Euler bends^31^ as access waveguides for increased effective interaction length are adopted to couple the incident Gaussian light from the single-mode fiber (SMF) to the WGMs in the microring resonator. Hence, such integrated emitters, which are significant to miniaturization, perform the same function as traditional diffractive optical elements (for example, spatial light modulators) for converting Gaussian light to optical vortices. Instead of using well-studied optical vortices that carry OAM^29^, here, we focus on two specific cylindrical vector beams (polarization vortices), which are generated by the integrated optical vortex emitters, that is, TM_01_ and TE_01_ modes, for the fiber vector eigenmode multiplexing transmission. More details about the device principle, design and fabrication are given in the Supplementary Information.

## Results and discussion

### Characterization of integrated optical vortex emitters

**Figure 2:**
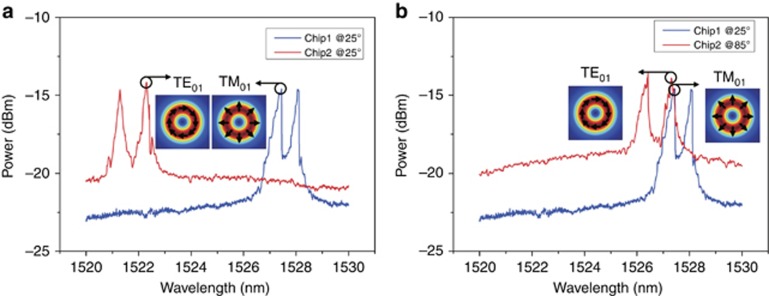
Measured radiation spectra from two chips showing mode splitting and thermal tuning. Mode splitting manifests in the splitting of the radiation spectrum, with the shorter and longer wavelength resonances associated with the TM_01_ and TE_01_ modes, respectively. Thermal tuning by heating the chip leads to a redshift of the radiation spectrum. The redshifted wavelength of the TE_01_ mode from Chip2 is identical to the wavelength of the TM_01_ mode from Chip1 for the fiber vector eigenmode multiplexing transmission. (**a**) Radiation spectra of two chips at room temperature of 25 °C. (**b**) Radiation spectra of Chip1 at 25 °C and Chip2 at 85 °C.

First, we characterize the performance of the fabricated integrated optical vortex emitters (Supplementary Information). We consider the case when the wavelength-dependent azimuthal order of the WGM in the microring resonator is equal to the number of etched grating elements in the inner sidewall of the microring resonator. Note that there is a mode splitting, which originates from the Bragg reflection-induced strong cross-coupling between the degenerate clockwise and counter-clockwise travelling WGMs inside the microring resonator (Supplementary Information). The mode splitting causes the splitting of the radiation spectrum, which is measured by scanning the incident laser wavelength. We fabricate two integrated optical vortex emitters (Chip1 and Chip2) and measure their radiation spectra, as shown in Figure 2. The radius of the two chips is 7.5 μm. One can clearly see the splitting of the radiation spectrum. The shorter and longer wavelength resonances of the split spectrum are associated with the radially polarized mode (TM_01_) and the azimuthally polarized mode (TE_01_), respectively. As shown in Figure 2a, the original radiation wavelengths of the two chips are different. To facilitate identical wavelengths of the TM_01_ mode from Chip1 and the TE_01_ mode from Chip2 for the fiber vector eigenmode multiplexing transmission, thermal tuning of Chip2 is performed to shift its radiation wavelength (Supplementary Information), as shown in Figure 2b.

**Figure 3:**
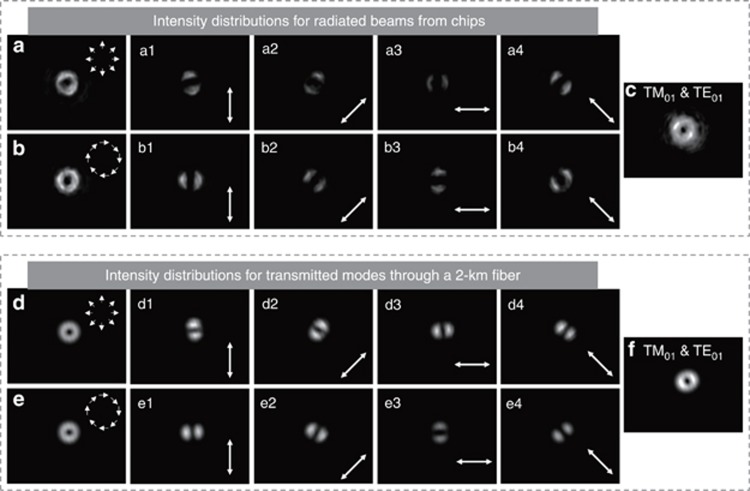
Measured intensity distributions for the fiber vector eigenmode (TM_01_, TE_01_) generation from chips and multiplexing transmission through a 2-km LCF. (**a**, **a**1–**a**4, **b**, **b**1–**b**4 and **c**) Radiated beams from the chips. (**d**, **d**1–**d**4, **e**, **e**1–**e**4 and **f**) Transmitted modes through a 2-km fiber. (**a** and **d**) TM_01_ mode (radially polarized mode). Insets illustrate spatial variant polarization distributions. (**a**1–**a**4 and **d**1–**d**4) TM_01_ mode after a rotating polarizer at different axis directions of 90 (**a**1 and **d**1), 45 (**a**2 and **d**2), 0 (**a**3 and **d**3), and −45 degree (**a**4 and **d**4). (**b** and **e**) TE_01_ mode (azimuthally polarized mode). Insets illustrate spatial variant polarization distributions. (**b**1–**b**4 and **e**1–**e**4) TE_01_ mode after a rotating polarizer at different axis directions of 0 (**b**1 and **e**1), 45 (**b**2 and **e**2), 90 (**b**3 and **e**3), and 135 degree (**b**4 and **e**4). (**c** and **f**) Multiplexed TM_01_ and TE_01_ modes after transmission through the fiber.

Then, we verify the generation of TM_01_ and TE_01_ modes using the two chips. The measured near-field intensity distributions of the TM_01_ mode from Chip1 and the TE_01_ mode from Chip2 are shown in Figure 3a and 3b, respectively. One can clearly see the doughnut intensity profiles due to polarization singularity with an undefined polarization and the resultant null intensity at the beam center. After passing through a rotating polarizer, the measured near-field intensity distributions of the TM_01_ and TE_01_ modes are shown in Figure 3a1–3a4 and Figure 3b1–3b4 respectively. According to the directions of the polarizer axis marked with white arrows and the lobe-like intensity profiles (Supplementary Movies S1 and S2), one can confirm that the radiated beams from Chip1 and Chip2 are TM_01_ and TE_01_ modes, respectively. The intensity distribution of the multiplexed TM_01_ and TE_01_ modes is shown in Figure 3c. The radiated beams from the two chips are sent to a 2-km LCF link. After the fiber transmission, the measured intensity distributions of the TM_01_ mode, TM_01_ mode through a rotating polarizer (Supplementary Movie S3), TE_01_ mode, TE_01_ mode through a rotating polarizer (Supplementary Movie S4), and multiplexing of the TM_01_ and TE_01_ modes are shown in Figure 3d, 3d1–3d4, 3e, 3e1–3e4 and 3f, respectively. The observed intensity distributions indicate good quality of the TM_01_ and TE_01_ modes, which are radiated from the two chips and transmitted through a km-scale LCF. The slight tilt of the measured intensity distributions after the rotating polarizer may be due to the unwanted spurious components being radiated from the chips or excited during the fiber transmission. In addition to the rotating polarizer, a mode decomposition tool based on q-plates can also be used to verify the TM_01_ and TE_01_ modes (vector modes)^32^.

### Performance of data-carrying fiber vector eigenmode multiplexing transmission

**Figure 4:**
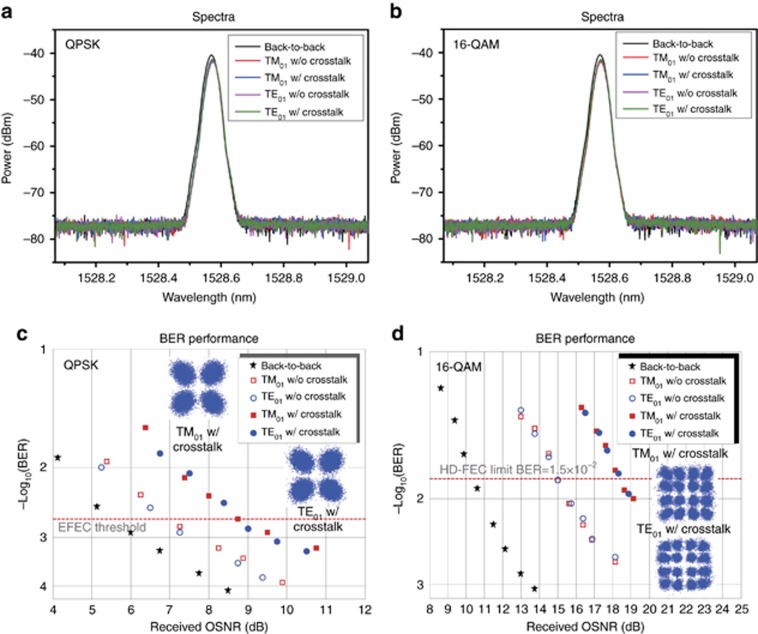
Measured system performance of the data-carrying fiber vector eigenmode multiplexing transmission through a 2-km LCF. (**a** and **b**) Measured spectra of the demultiplexed TM_01_ and TE_01_ modes. (**c** and **d**) Measured bit-error rate (BER) versus received optical signal-to-noise ratio (OSNR) for the demultiplexed TM_01_ and TE_01_ modes. Insets show constellations of QPSK (**c**) and 16-QAM (**d**). (**a** and **c**) 10-Gbit s^−1^ QPSK signals. (**b** and **d**) 20-Gbit s^−1^ 16-QAM signals. The crosstalk between TM_01_ and TE_01_ modes at the output of the LCF is approximately −16 dB. EFEC: enhanced forward-error correction. HD-FEC: hard-decision forward-error correction. w/o crosstalk: without crosstalk. w/ crosstalk: with crosstalk.

We further set up an experimental configuration (Supplementary Information) to study the system performance of data-carrying fiber vector eigenmode (TM_01_, TE_01_) multiplexing transmission through a 2-km LCF. The TM_01_ and TE_01_ modes emitted from Chip1 and Chip2 carry 10-Gbit s^−1^ QPSK or 20-Gbit s^−1^ 16-QAM signals. The measured spectra of the demultiplexed TM_01_ and TE_01_ modes after the QPSK or 16-QAM-carrying fiber vector eigenmode multiplexing transmission are shown in Figure 4a and 4b, respectively. These spectra overlap with one another, occupying the same bandwidth. The measured bit-error rate (BER) curves as a function of the received optical signal-to-noise ratio (OSNR) for the QPSK or 16-QAM-carrying fiber vector eigenmode multiplexing transmission are shown in Figure 4c and 4d, respectively. Insets in Figure 4c and 4d depict the constellations of QPSK and 16-QAM, respectively. In the experiment, polarization controllers on a large-core fiber (PC-LCF) are employed to assist the mitigation of the crosstalk between the TM_01_ and TE_01_ modes (Supplementary Information). The measured mode crosstalk at the output of LCF is approximately −16 dB. The measured TM_01_- or TE_01_-only results without crosstalk (w/o crosstalk) are also depicted in Figure 4 for comparison with those with crosstalk (w/ crosstalk). For the 10-Gbit s^−1^ QPSK signals, the measured OSNR penalties of the demultiplexed TM_01_ and TE_01_ modes are approximately 1.5 dB w/o crosstalk and 3.2 dB w/ crosstalk at a BER of 2 × 10^−3^, which is the enhanced forward error correction (EFEC) threshold. For the 20-Gbit s^−1^ 16-QAM signals, the measured OSNR penalties of demultiplexed TM_01_ and TE_01_ modes are approximately 4.5 dB w/o crosstalk and 7.7 dB w/ crosstalk at a BER of 1.5 × 10^−2^, which is the limit for a hard-decision forward-error correction (HD-FEC) with a 20% overhead. The obtained results indicate a successful implementation of a km-scale data-carrying fiber vector eigenmode multiplexing transmission seeded by integrated optical vortex emitters with a favorable performance.

## Conclusions

In summary, by exploiting the optical polarization vortices generated from integrated optical vortex emitters, we report data-carrying fiber vector eigenmode multiplexing transmission through a 2-km LCF. Such fiber vector eigenmode multiplexing transmission can open up added capacity scaling opportunities by directly using the multiple vector eigenmodes of a specially designed multi-mode fiber, paving an alternative yet straightforward way for fiber-based spatial mode multiplexing. The integrated optical vortex emitters can provide truly compact solutions to replace bulky diffractive optical elements for the generation of fiber-eigenmode-like optical polarization vortices. Beyond the proof-of-concept demonstration on two fiber vector eigenmode (TM_01_ and TE_01_) multiplexing transmission seeded by silicon microring resonators with inner sidewall angular gratings, there are significant challenges and potential solutions towards a large number of fiber vector eigenmode multiplexing transmission.

Though the proper structural design of the LCF and the use of PC-LCF led to reduced mode crosstalk and resultant two fiber vector eigenmode multiplexing transmission, a specialty fiber that supports many fiber vector eigenmode multiplexing transmission with low-level crosstalk is still a challenge. Conventional weakly guiding multi-mode fibers support many vector eigenmodes, which, however, suffer strong mode coupling due to their similar effective refractive indices, especially in the same mode group. Fortunately, the high-index-contrast ring-core/air-core fiber can obtain negligible mode crosstalk by lifting the degeneracy between the neighboring fiber vector eigenmodes^33, 34^ and achieving an effective refractive index difference larger than 10^−4^, which is also the typical value of birefringence in the polarization-maintaining fiber (PMF). Moreover, the elliptical-core fiber can also help the mitigation of mode crosstalk^35, 36, 37^. Hence, the optimized design of a specialty fiber borrowing the ideas from the ring-core/air-core fiber, elliptical-core fiber, and PMF may enable full-vectorial multiple fiber eigenmode multiplexing transmission that is free of multiple-input multiple-output (MIMO) digital signal processing.

Though the silicon microring resonators with inner sidewall angular gratings generate fiber-eigenmode-like TM_01_ and TE_01_ vector modes, the (de)multiplexer of vector modes using a single photonic integrated chip is another challenge. Fortunately, integrated building blocks, including chip-scale Mach-Zehnder interferometers, phase shifters, and 2-dimensional (2D) gratings, can facilitate flexible spatial amplitude/phase/polarization manipulation^38, 39^. By employing a 2D grating array arranged in a circle, with each 2D grating manipulating its local polarization^38^, it is possible to build up a vector mode generator and (de)multiplexer (TM_01_, TE_01_) on a single photonic integrated chip. Moreover, high-order vector modes may also be generated and (de)multiplexed using a single photonic integrated chip composed of the building blocks with an appropriate layout for the desired spatial amplitude/phase/polarization manipulation. Hence, the optimized design of monolithic integration that properly incorporates building blocks may enable flexible arbitrary vector mode manipulation for full-vectorial multiple fiber eigenmode multiplexing transmission.

This can be the future envisaged roadmap toward practical applications in vector mode communications. It holds the potential to develop many data-carrying fiber vector eigenmode multiplexing transmission seeded by compact photonic integrated circuits.

## Author contributions

JW developed the concept and conceived the experiment. XC conceived the device design. XC, CK and MS fabricated the device. CD and QM provided the fiber. JL, SML, LZ, ADW and JW constructed the experiment. JL, SML, SC and JW performed the theoretical analyses, acquired the experimental data, and carried out the data analysis. All authors contributed to writing. JW finalized the paper. JW and XC supervised the project.
