# Supplementary material for: Direct fiber vector eigenmode multiplexing transmission seeded by integrated optical vortex emitters
Source: Light Sci Appl. 2018 Mar 9;7:17148–. doi: 10.1038/lsa.2017.148 (PMC6060045; doi:10.1038/lsa.2017.148)
Supplement: Supplementary Information [file lsa2017148x1.docx]

**Supplementary Information for**

Direct Fiber Vector Eigenmode Multiplexing Transmission Seeded by Integrated Optical Vortex Emitters

Jun Liu^1,*^, Shi-Mao Li^2,*^, Long Zhu^1^, An-Dong Wang^1^, Shi Chen^1^, Charalambos Klitis^3^, Cheng Du^4^, Qi Mo^1,4^, Marc Sorel^3^, Si-Yuan Yu^2^, Xin-Lun Cai^2,†^ and Jian Wang^1,†^

^1^ Wuhan National Laboratory for Optoelectronics, School of Optical and Electronic Information, Huazhong University of Science and Technology, Wuhan 430074, Hubei, China.

^2^ State Key Laboratory of Optoelectronic Materials and Technologies and School of Physics and Engineering, Sun Yatsen University, Guangzhou 510275, China.

^3^ School of Engineering, University of Glasgow, Rankine Building, Oakfield Avenue, Glasgow G12 8LT, UK.

^4^ Fiberhome Telecommunication Technologies Co. Ltd, Wuhan 430074, China.

* These authors contributed equally to this work.

† Correspondence to: jwang@hust.edu.cn (J.W.); caixlun5@mail.sysu.edu.cn (X.C.)

1. Device Design and Fabrication

Two integrated optical vortex emitters, i.e. silicon microring resonators with inner sidewall etched as angular gratings, are fabricated on a silicon-on-insulator (SOI) wafer with a 220-nm-thick silicon core and a 2-$\mu$m-thick buried oxide layer. The microring resonator is defined with a radius of 7.5 μm and a waveguide width of 500 nm. The period of the grating is about 620 nm, corresponding to one wavelength in the microring resonator to form a second order grating for the vacuum wavelength of 1550 nm. The grating elements protrude from the inner sidewall of the microring waveguide by as much as 60 nm, and their width is 60 nm. To get significant coupling to the whispering gallery modes (WGMs) of the microring resonator, we use the so-called Euler bends^1^ as access waveguide to increase the effective interaction length. The distance between the access waveguide and the microring resonator increases slowly from 150 nm. The width of the access waveguide is 410 nm to meet the phase matching condition of the bend-coupling. To reduce the coupling loss between the fiber and waveguide facet as well as the Fabry-Perot resonance effects caused by optical reflection from the facets, an end coupling structure which consists of inversely tapered waveguides is fabricated at both ends of the access waveguide. The designed structures are fabricated using an electron-beam lithography (EBL) which defines the photonic integrated device pattern in a negative e-beam resist, followed by an inductively coupled plasma (ICP) etching process to transfer the photonic integrated device pattern into the silicon layer. After the etching process, a 300-nm-thick silicon dioxide layer is deposited onto the wafer to encapsulate the silicon waveguides. Fig. S1 illustrates the 3D-sketch of the designed and fabricated integrated optical vortex emitters.


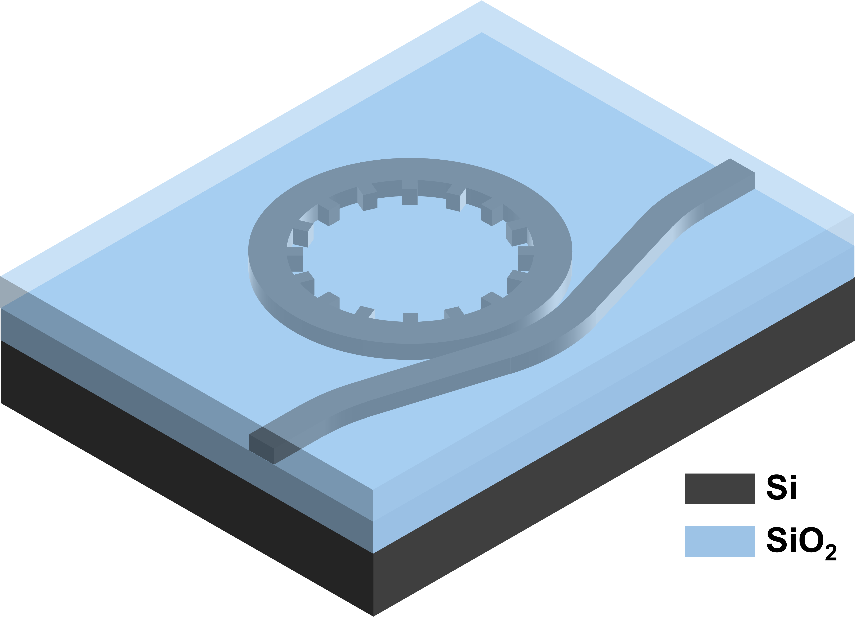


**Figure S1 |** 3D-sketch of the deigned and fabricated integrated optical vortex emitters (silicon microring resonators with inner sidewall etched as gratings).

2. Device Characterization

An experimental setup for characterizing the performance of the fabricated integrated optical vortex emitters is shown in Fig. S2a. A polarization controller (PC) is used to adjust the polarization state of light beam from the tunable laser for efficient mode excitation in the access waveguide of the emitter chip. The light with optimized polarization is coupled into the chip through the access waveguide by a tapered lens fiber tip. The output light from the access waveguide of the emitter chip is collected by another tapered lens fiber tip and the power is measured by a power meter A. Meanwhile, the inner sidewall etched angular gratings of the microring resonator can extract the confined WGMs in the microring resonator into vertically radiated beams, which are collimated by a 40X objective lens and the power is measured by a free-space power meter B. Shown in Fig. S2b is the measured typical radiation spectrum of the fabricated emitter chip (Chip1) by scanning the wavelength of the tunable laser from 1520 to 1580 nm. The radiated beams are optical vector vortices. Different resonances in the radiation spectrum correspond to different optical vector vortices. The splitting of the radiation spectrum is due to the mode splitting. The shorter and longer wavelength resonances of the split spectrum are associated with radially polarized mode (TM_01_) and azimuthally polarized mode (TE_01_), respectively, as marked in Fig. S2b.


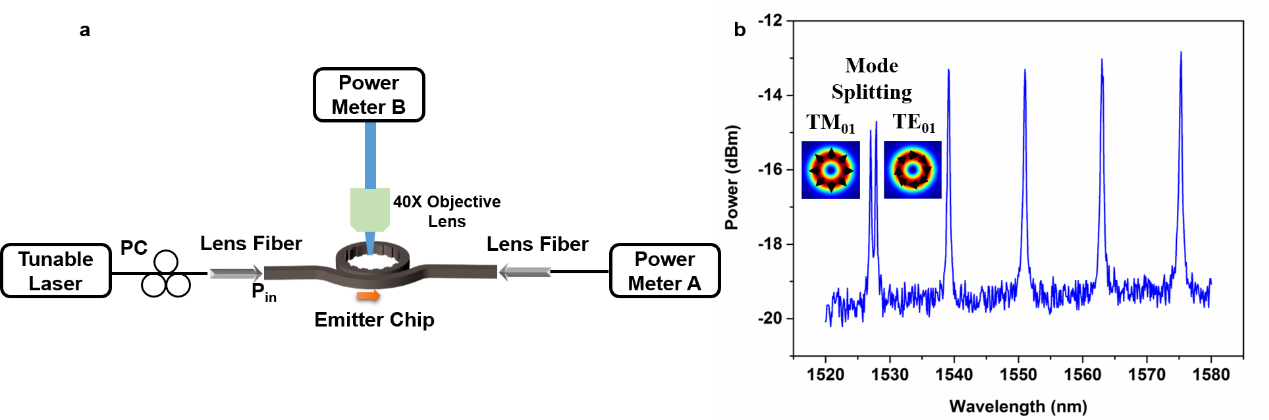


**Figure S2 |** Device characterization. (**a**) Experimental setup for characterizing the performance of the fabricated emitter chips. PC: polarization controller. (**b**) Measured radiation spectrum of the fabricated emitter chip (Chip1) by scanning the wavelength of the tunable laser from 1520 to 1580 nm.

3. Design Parameters of Large-Core Fiber (LCF)

Fig. S3a and S3b shows the relative refractive index profile and cross-section view of the designed large-core fiber (LCF), which is a circular core optical fiber with a step index profile. The radii of the fiber core and cladding are set to r_core_=6.35 $\mu m$ and r_cladding_=62.5 $\mu m$, respectively. The relative refractive index difference ($\Delta=(n_{1}-n_{2})/n_{2}$) between the fiber core ($n_{1}$) and cladding ($n_{2}$) is $\Delta$=0.377%. The simulated intensity profiles of TM_01_ and TE_01_ fiber vector eigenmodes are shown in Fig. S3c and S3e, respectively. The TM_01_ and TE_01_ fiber vector eigenmodes are radially and azimuthally polarized modes, respectively, both having undetermined polarization and resultant null intensity at the beam center. Fig. S3d and S3f plots the corresponding 3D intensity profiles of the TM_01_ and TE_01_ fiber vector eigenmodes. One can clearly see the doughnut intensity profiles due to polarization singularity at the beam center.


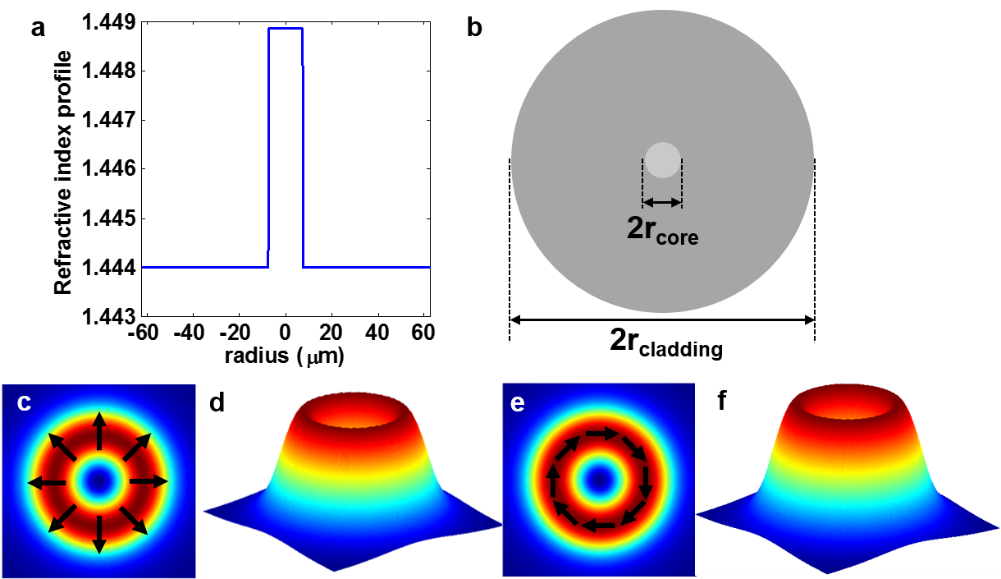


**Figure S3 |** **Design parameters of the large-core fiber (LCF).** (**a**) Relative refractive index profile. (**b**) Cross-section view. (**c**) TM_01_ fiber vector eigenmode with doughnut intensity profile and radially polarized field. (**d**) 3D intensity profile of TM_01_ mode. (**e**) TE_01_ fiber vector eigenmode with doughnut intensity profile and azimuthally polarized field. (**f**) 3D intensity profile of TE_01_ mode.

4. Experimental Configuration for Data-Carrying Fiber Vector Eigenmodes Multiplexing Transmission

The detailed experimental configuration for km-scale data-carrying fiber vector eigenmodes multiplexing transmission seeded by integrated optical vortex emitters is shown in Fig. S4. The experimental configuration consists of five parts, i.e. transmitter, multiplexing of chip-generated eigenmodes (fiber-eigenmodes-like polarization vortices), 2-km LCF transmission link, demultiplexing of two eigenmodes (TM_01_ and TE_01_ fiber vector eigenmodes), and receiver. At the transmitter side, a 10-Gbit s^-1^ quadrature phase-shift keying (QPSK) or 20-Gbit s^-1^ 16-ary quadrature amplitude modulation (16-QAM) signal is generated by a tunable laser followed by an optical in-phase/quadrature (I/Q) modulator, which is fed by an arbitrary waveform generator (AWG) with programmed 5-Gbaud QPSK/16-QAM electric signal. The 10-Gbit s^-1^ QPSK or 20-Gbit s^-1^ 16-QAM signal is amplified by an erbium-doped fiber amplifier (EDFA) followed by a tunable filter to remove the amplified spontaneous emission (ASE) noise. In the multiplexing part, before coupling into the input access waveguide of the microring resonator, the signal is divided by an optical coupler (OC) into two paths, relatively delayed by a piece of single-mode fiber (SMF) for decorrelation, and amplified a second time by EDFAs. Polarization controllers (PCs) are used to optimize the polarization state of light for efficient coupling into the access waveguide of the emitter chips. The power is monitored by a power meter placed at the output port of the access waveguide. The desired optical vector vortex modes, i.e. radially polarized TM_01_ mode and azimuthally polarized TE_01_ mode, are vertically excited from the emitter chips when the wavelength of the tunable laser is appropriately adjusted to be aligned to the corresponding resonance wavelengths of TM_01_ and TE_01_ modes. Note that the radiation spectra of two emitter chips (Chip1, Chip2) are not the same at room temperature which can be briefly explained as follows. In the fabrication of the two emitter chips, deviations from the designed microring structure can happen and the fabrication environment cannot be completely uniform, therefore, there is an inevitable shift of the radiation spectra between the two emitter chips. To get identical wavelengths of TM_01_ mode from Chip1 and TE_01_ mode from Chip2 for fiber vector eigenmodes multiplexing transmission, thermal tuning is employed to shift the radiation spectrum by heating the emitter chip. The emitted data-carrying TM_01_ and TE_01_ modes from two chips with the same wavelength are collimated by two 40X objective lenses and combined together by a beam splitter (BS). The two multiplexed TM_01_ and TE_01_ modes are coupled into the LCF by a 10X objective lens. After propagating through a 2-km LCF, the output beams are collimated again by a 20X objective lens. Two polarization controllers on LCF (PC-LCF) are used to mitigate the mode crosstalk. In the demultiplexing part, for easy separation a linear polarizer (Pol.) is used to transform TM_01_ and TE_01_ modes to corresponding LP_11_-like beams (LP_11a_ and LP_11b_), which are then converted to a Gaussian-like beam by using a spatial light modulator (SLM) loaded with a specific phase pattern. A camera is used in the multiplexing and demultiplexing parts to observe the intensity distributions of emitted light beams from the chips and transmitted light beams after the 2-km LCF. At the receiver side (coherent detection), the converted Gaussian-like beam is coupled into an SMF, amplitude by a third EDFA, and ASE-noise-suppressed by a tunable filter. A variable optical attenuator (VOA) followed by a fourth EDFA are then used to adjust the received optical signal-to-noise ratio (OSNR) for bit-error rate (BER) performance measurement. Another tunable laser serves as a local oscillator to mix with the received signal by an optical hybrid, the outputs of which are sent to an oscilloscope followed by offline digital signal processing.


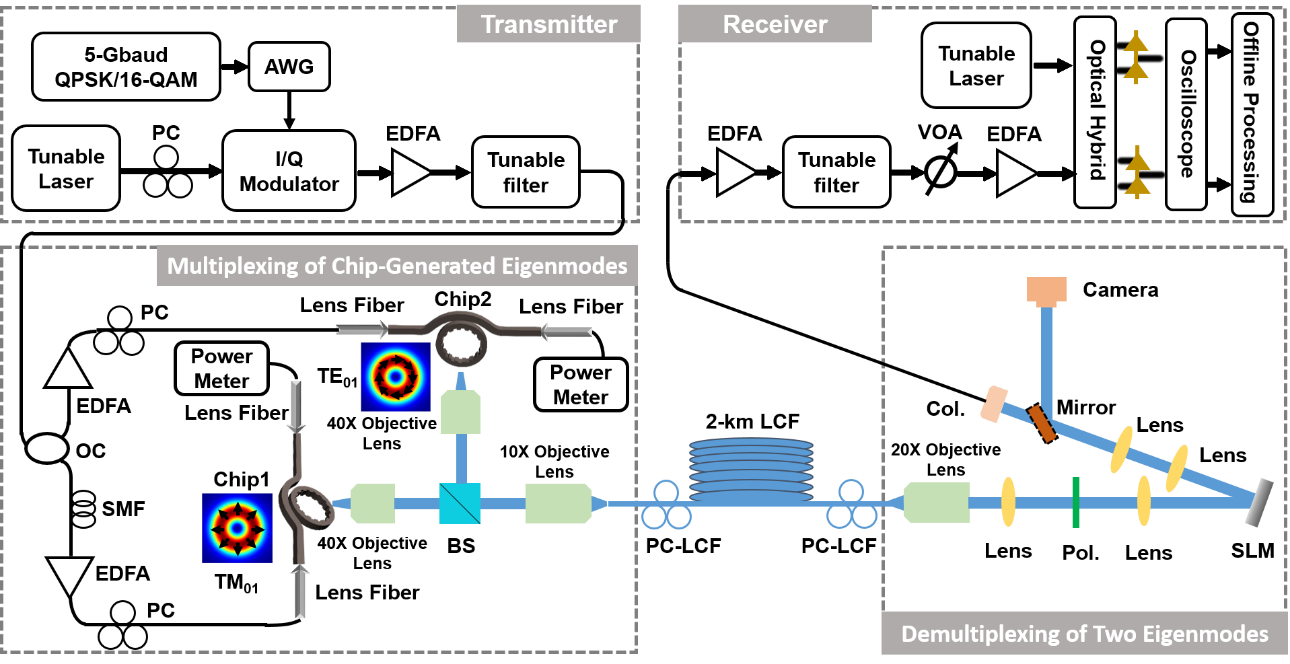


**Figure S4 |** Experimental configuration for km-scale data-carrying fiber vector eigenmodes multiplexing transmission seeded by integrated optical vortex emitters. The configuration consists of five parts (transmitter, multiplexing of chip-generated eigenmodes, 2-km large-core fiber (LCF) transmission link, demultiplexing of two eigenmodes, and receiver). PC: polarization controller; I/Q: in-phase/quadrature; QPSK: quadrature phase-shift keying; QAM: quadrature amplitude modulation; AWG: arbitrary waveform generator; EDFA: erbium-doped fiber amplifier; OC: optical coupler; SMF: single-mode fiber; BS: beam splitter; LCF: large-core fiber; PC-LCF: polarization controller on large-core fiber; Pol.: polarizer; SLM: spatial light modulator; Col.: collimator; VOA: variable optical attenuator.

5. Mode Crosstalk Mitigation Assisted by Polarization Controllers on Large-Core Fiber (PC-LCF)

In the experiments, we are able to control the mode coupling in the LCF by using polarization paddle controllers. The use of PC-LCF is equivalent to mitigating mode crosstalk using multiple-input multiple-output (MIMO) digital signal processing. One can change the “channel matrix” of the LCF until it is approximately diagonal by adjusting the PC-LCF. A diagonal “channel matrix” means little mode coupling. Hence, the employed PC-LCF assists the mitigation of crosstalk between TM_01_ and TE_01_ modes. The measured mode crosstalk between TM_01_ and TE_01_ modes at the output of LCF is about -16 dB.

6. Radially and Azimuthally Polarized Fields in the Silicon Microring Resonator

There are two known states of polarizations (SOPs) associated with WGMs in the microring resonator, i.e. the quasi-transverse electric (quasi-TE) mode with transverse electric-field dominated by $E_{r}$ (radial field in Fig. S5a) and the quasi-transverse magnetic (quasi-TM) mode with transverse electric-field dominated by $E_{z}$. Here, we consider the fundamental quasi-TE mode. Actually, the longitudinal component, $E_{\varphi}$ (azimuthal field in Fig. S5a), also exists in channel waveguide as it originates from the spatial derivative of the transverse field, and for the quasi-TE mode it can be approximately expressed as^2^

$E_{\varphi}\approx\frac{1}{j\beta}\frac{\partial E_{r}}{\partial r}$ (1)

where $\beta$ is the propagation constant. Eq. (1) shows that the longitudinal component, $E_{\varphi}$, is always in quadrature phase ($\pi$/2) with the transverse electric field. Specifically, in high-index-contrast silicon waveguide, the magnitude of the $E_{\varphi}$ component can be comparable to the transverse electric field due to the ultra-tight optical confinement. There are no exact solutions for the electric field of channel waveguide, while it can be calculated by using finite difference eigenmode (FDE) method. Fig. S5b and S5c shows the $E_{r}$ and $E_{\varphi}$ components of the fundamental quasi-TE mode at a wavelength of 1550 nm supported by a silicon microring resonator with a 7.5 $\mu m$ radius and 500$\times$220 nm^2^ (width $\times$ height) dimensions surrounded by a silicon dioxide (SiO_2_) layer. The largest $E_{\varphi}$ amplitude occurs near the waveguide sidewalls since it is maximized where the transverse spatial derivative is maximized. Comparing $E_{r}$ and $E_{\varphi}$ (Fig. S5d) shows that in the inner-sidewall of the silicon microring resonator where the grating locates the evanescent of $E_{\varphi}$ is comparable to that of the transverse electric field $E_{r}$ component, meaning that both radially and azimuthally polarized fields can be achieved.


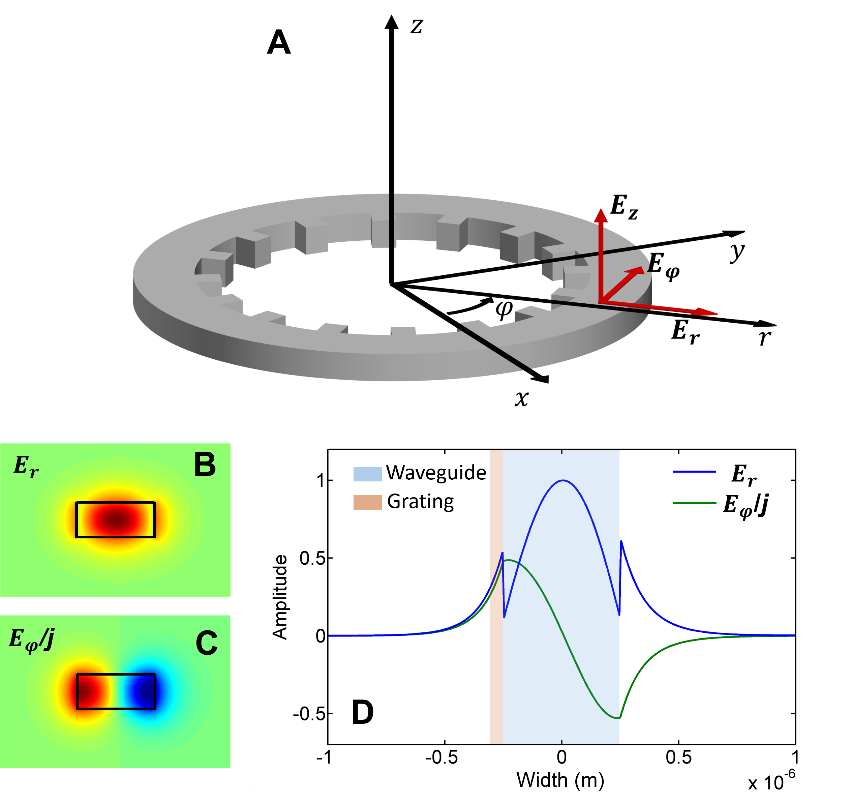


**c**

**b**

**d**

**a**

**Figure S5 |** (**a**) Schematic illustration of electric field in the microring resonator in a cylindrical coordinate system. (**b**) $E_{r}$ (radial field) and (**c**) $E_{\varphi}$ (azimuthal field) components of the fundamental quasi-TE mode supported by a silicon microring resonator with a radius of 7.5-$\mu m$, cross-section dimensions of 500$\times$220 nm^2^ and a silicon dioxide (SiO_2_) cladding. (**d**) Comparison between $E_{r}$ and $E_{\varphi}$.

7. Mode Splitting of WGMs in the Silicon Microring Resonator and Generation of TM_01_ and TE_01_ Modes

The radiated beams from the silicon microring resonator with etched angular gratings are optical vector vortices with a topological Pancharatnam charge of $p$-$q$, where $p$ is the azimuthal order of the WGM involved or the number of optical periods around the microring resonator, and $q$ is the number of etched grating elements of the microring resonator^3^. At the wavelength where $p$ equal to $q$, the travelling WGM will be split into two standing-wave-modes (SWMs) (i.e. symmetric mode and antisymmetric mode) due to strong Bragg reflection. The form of the symmetric mode and antisymmetric mode and their frequency splitting can be derived from transfer matrix analysis^4^ or coupled mode theory^5,6^.

The microring resonator supports two degenerate WGMs at each resonance wavelength with the same field distributions but opposite propagation directions. As illustrated in Fig. S6a, they are clockwise (CW) modes and counter-clockwise (CCW) modes. Such degeneracy will be lifted to split the resonance into doublets due to the perturbation in the mode volume^7^. Here, the second-order grating is employed and the period satisfies the condition of

$\Lambda\approx\frac{2\pi}{\beta}$ (2)

where $\beta$ is the propagation constant of light in the waveguide. In the case of weak harmonic modulation of the microring waveguide width and subject to the condition Eq. (2), the propagation of a CCW-WGM ($\beta$) along the microring is accompanied by some of the first- and second-order diffracted waves of a CW-WGM ($-\beta$), which has the same field distribution as the original CCW-WGM but travels in the opposite direction, as illustrated in Fig. S6b. The incident light $E_{in}$ only excites the CCW traveling mode $E_{CCW}$, and the CW traveling mode $E_{CW}$ relates with $E_{CCW}$ by a mutual coupling coefficient $\gamma$. For the two modes, the decay rate can be expressed as^8^

$\frac{dE_{CCW}}{dt}=\left[ i\left( \omega-\omega_{0} \right)-\frac{1}{\tau} \right]E_{CCW}+\kappa E_{in}+i\gamma E_{CW}$ (3a)

$\frac{dE_{CW}}{dt}=\left[ i\left( \omega-\omega_{0} \right)-\frac{1}{\tau} \right]E_{CW}+i\gamma E_{CCW}$ (3b)

where $\omega$ is the incident frequency, $\omega_{0}$ is the resonant frequency of the two degenerate modes, $\tau$ is the total photon lifetime, and $\kappa$ is the coupling efficiency between the access waveguide and ring resonator. The two degenerate modes will be superimposed symmetrically or antisymmetrically to form SWMs, which can be described by

$E_{SM}=E_{CCW}+E_{CW}$ (4)

or

$E_{ASM}=E_{CCW}-E_{CW}$ (5)

By combining Eq. (3) and Eq. (4), we obtain the decay rate of symmetric SWM as

$\frac{dE_{SM}}{dt}=\left[ i\left( \omega-\omega_{0} \right)-\frac{1}{\tau_{1}} \right]E_{SM}+\kappa E_{in}+i\gamma E_{SM}$ (6)

Similarly, for the antisymmetric SWM, the decay rate is

$\frac{dE_{ASM}}{dt}=\left[ i\left( \omega-\omega_{0} \right)-\frac{1}{\tau_{2}} \right]E_{ASM}+\kappa E_{in}-i\gamma E_{ASM}$ (7)

In general, the two SWMs have different scattering loss, thus the new-born eigenmodes have unequal photon lifetime ($\tau_{1}$, $\tau_{2}$). In the steady state, Eq. (6) and Eq. (7) can be respectively simplified as

$E_{SM}=\frac{\kappa E_{in}}{-i[\omega-{(\omega}_{0}-\gamma)]+\frac{1}{\tau_{1}}}$ (8)

and

$E_{ASM}=\frac{\kappa E_{in}}{-i[\omega-{(\omega}_{0}+\gamma)]+\frac{1}{\tau_{2}}}$ (9)

It is clear from Eqs. (8) and Eq. (9) that the symmetric mode (SM) resonates at $\omega=\omega_{0}-\gamma$, and the antisymmetric mode (ASM) resonates at $\omega=\omega_{0}+\gamma$, i.e. the new-born eigenmodes resonate at distinct wavelengths, and therefore a doublet can be seen in the transmission and radiation spectra (mode splitting induced spectrum splitting). It is noted that the amount of the doublet splitting, $2\gamma$, should be larger than the frequency linewidth of the WGM to resolve the splitting in the spectrum^9^. Indeed, the frequency linewidth of the WGM is large as the quality factor is deteriorated by the grating, however, the second-order diffraction of the angular grating, i.e. the reflection of the travelling WGM, is so strong that the amount of splitting is larger enough to overcome the frequency linewidth, introducing observable doublet in the spectrum.


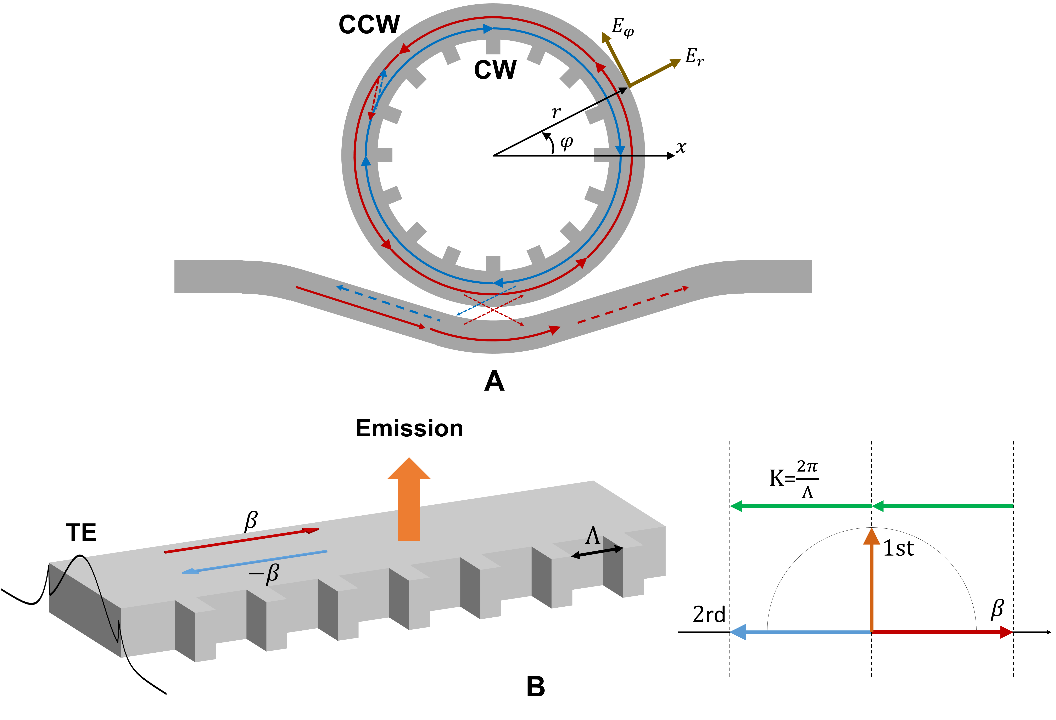


**b**

**a**

**Figure S6 |** (**a**) Schematic illustration of the coupled microring-grating system. Input light propagating from left to right in the bended waveguide $E_{in}$ excites the CCW traveling mode $E_{CCW}$ in the ring. $E_{CCW}$ couples the CW traveling mode $E_{CW}$ with coefficient $\gamma$ due to the Bragg grating along ring. (**b**) Schematic illustration and vector diagram of Bragg (second-order) resonance diffraction. Wave vector ($\beta$) of the incident wave is equal to the grating vector ($K$).

Fig. S7a shows a typical example of the radiation spectrum for a device with radius *R*=2.5 μm, number of grating elements $q$=24, and grating size of 150×150 nm^2^ simulated by FDTD, the doublet in which manifests the creation of SWMs. The right peak at longer wavelength represents the symmetric mode (SM), and the left peak at shorter wavelength represents the antisymmetric mode (ASM). The sine and cosine SWMs are self-adjusted so that the local equally distributed grating elements are placed in a node (antinode) of the mode, e.g. $E_{\varphi}$ in the ASM locates the grating at the node (Fig. S7b) and $E_{\varphi}$ in the SM locates the grating at the antinode (Fig. S7c). There is one important characteristic of the SWMs, i.e. the node of $E_{\varphi}$ always aligns to the antinode of $E_{r}$ due to the π/2 phase difference between them. Consequently, for the ASM, $E_{\varphi}$ locates the grating at the node with oppsite directions at each side of the elements and thus is suppressed by destructive interference, while $E_{r}$ locates the grating at the antinode with the same directions in the whole area of the elements (the red arrow in Fig. S7b) and thus is radiated to free-space to form a cylindrical vector vortex beam, which is a radially polarized beam (TM_01_). In contrast, for the SM, $E_{r}$ is suppressed while $E_{\varphi}$ is radiated, which is an azimuthally polarized beam (TE_01_) (Fig. S7c).

We define the confinement factor ($\Gamma$) as the power fraction of the mode that is in the waveguide region (silicon microring with angular gratings). Note that $E_{\varphi}$ is distributed more near the sidewalls of the microring resonator with better overlap with the grating. The confinement factor of the SM $(\Gamma_{SM}=0.854$) is slightly larger than that of the ASM ($\Gamma_{ASM}=0.842$), indicating that the associated effective refractive index, $n_{SM}$, is larger than $n_{ASM}$. A relatively large effective refractive index is corresponding to a longer resonance wavelength. That is, the distributions of SM and ASM with respect to the grating also determine their locations in the doublet, i.e. the ASM (TM_01_ mode) always resonates at the shorter wavelength, while the SM (TE_01_ mode) at another longer wavelength (Fig. S7a).


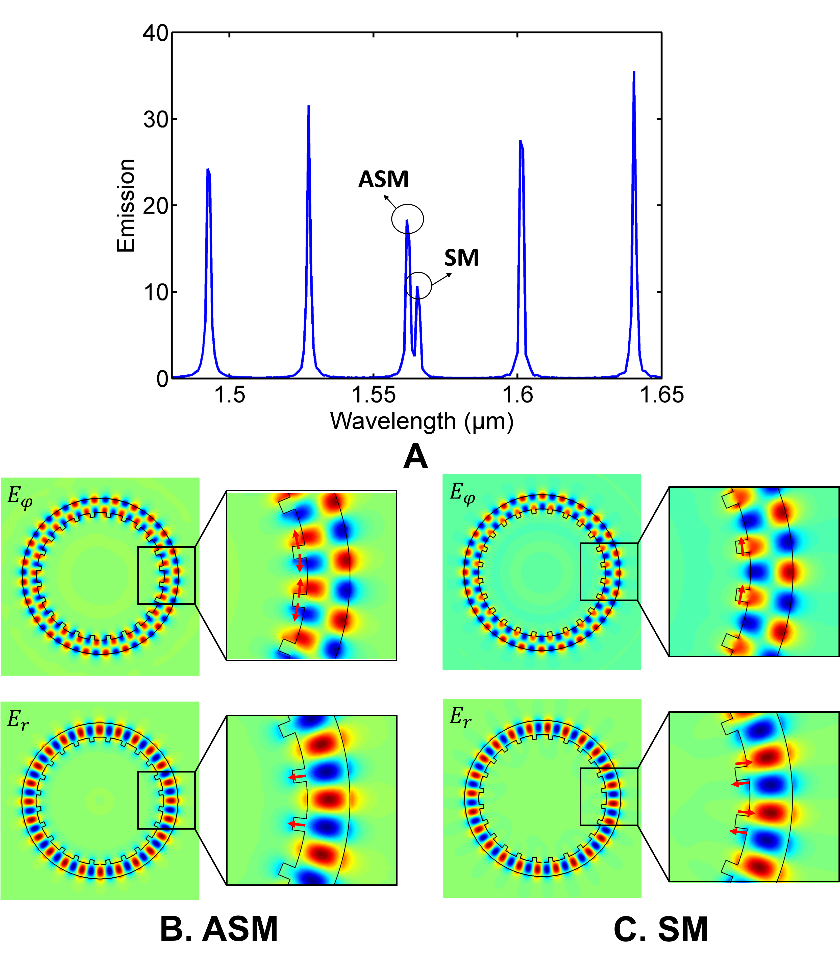


**c**

**b**

**a**

**Figure S7 |** FDTD simulation results for a device with radius *R*=2.5 μm, number of grating elements $q$=24, and grating size of 150×150 nm^2^. (**a**) Radiation spectrum with distinct wavelength splitting (doublets) due to mode splitting. (**b, c**) Azimuthal (up: $E_{\varphi}$) and radial (bottom: $E_{r}$) components of electric field distribution of the antisymmetric mode (ASM) (**b**) and the symmetric mode (SM) (**c**). The insets in (**b**) and (**c**) show the zoom-in views of the electric field and the locations of the field nodes and antinodes with respect to grating elements. The red arrows show the directions of the electric field.

8. Additional Results of Thermal Tuning of the Chip

The wavelengths of the radiated TM_01_ mode from Chip1 and TE_01_ mode from Chip2 are different at room temperature of 25℃. To achieve identical wavelengths of TM_01_ and TE_01_ modes for fiber vector eigenmodes multiplexing transmission, Chip2 is heated to shift its radiation spectrum. We study in detail the thermal tuning effect of the emitter chip. Fig. S8a plots the measured radiation wavelengths of TM_01_ and TE_01_ modes from Chip2 as a function of the temperature, indicating a linear relationship between the increase of temperature and the resultant wavelength shift. Fig. S8b shows the measured adjustable radiation spectrum of Chip2 at different temperatures. One can clearly see that the wavelengths of TM_01_ and TE_01_ modes can be tuned to be identical by the thermal tuning of emitter chip.


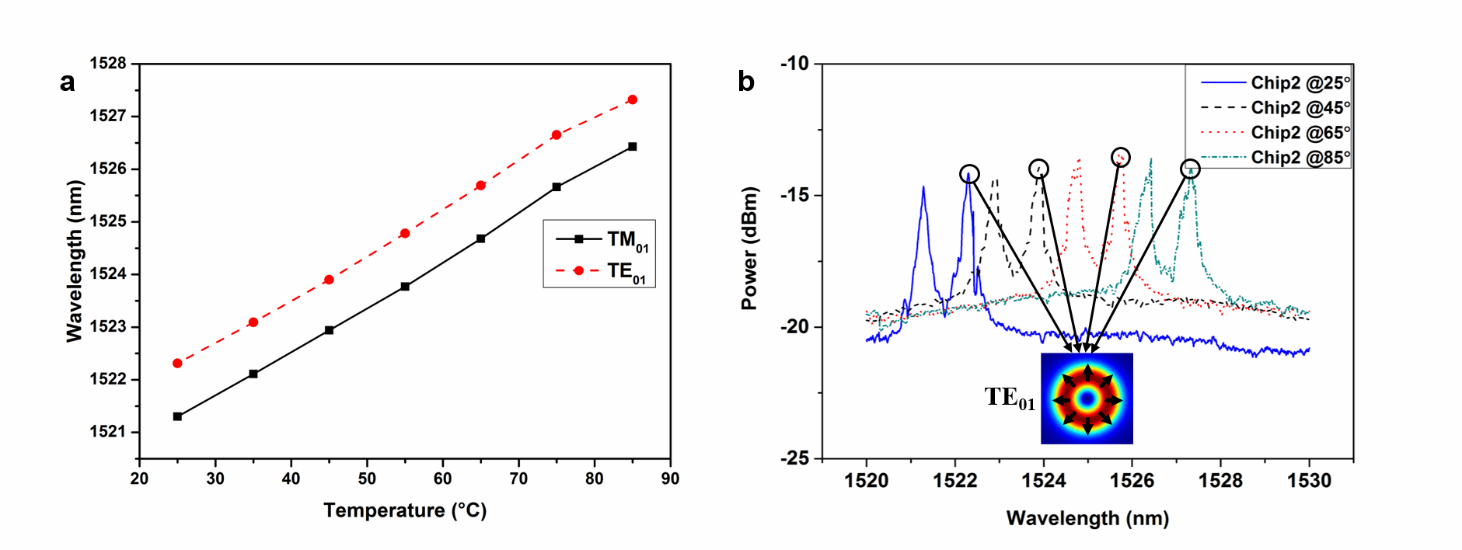


**Figure S8 |** Thermal tuning effect of the emitter chip. (**a**) Measured radiation wavelength shift of TM_01_ and TE_01_ modes of Chip2 versus temperature. (**b**) Measured adjustable radiation spectrum of Chip2 at different temperatures.

9. Additional Results of Demultiplexing of TM_01_ and TE_01_ Modes

A polarizer and an SLM are employed to detect TM_01_ and TE_01_ modes. After the fiber vector eigenmodes multiplexing transmission through a 2-km LCF, the TM_01_ and TE_01_ modes (Fig. S9a and S9b) pass through a polarizer (horizontal polarizer axis) and are converted to different LP_11_-like modes (Fig. S9c and S9d). Fig. S9e and S9f depicts the phase patterns loaded to the SLM for the demodulation of LP_11_-like modes. The phase pattern in Fig. S9e (Fig. S9f) is formed by the superposition of a left-right 0/π (up-down 0/π) pattern and a uniform grating pattern in the dashed box. For the demultiplexing of TM_01_ mode emitted from Chip1, the phase pattern in Fig. S9e gives a bright spot at the beam center (back-converted Gaussian-like beam), as shown in Fig. S9g, which can be coupled into an SMF for detection. For the demultiplexing of TE_01_ mode emitted from Chip2, the phase pattern in Fig. S9f gives a back-converted Gaussian-like beam with a central bright spot sent to an SMF for detection. In contrast, when demultiplexing TM_01_ mode with the phase pattern in Fig. S9f and demultiplexing TE_01_ mode with the phase pattern in Fig. S9e, the measured intensity distributions are shown in Figs. S9h and S9i, respectively, which can be regarded as the crosstalk of demultiplexing. The null intensity at the beam center in Fig. S9h and S9i indicate low-level crosstalk of demultiplexing. For the multiplexed TM_01_ and TE_01_ modes, the measured intensity distributions for TM_01_ (phase pattern in Fig. S9e) and TE_01_ (phase pattern in Fig. S9f) demultiplexing with crosstalk are shown in Fig. S9k and S9l, respectively.


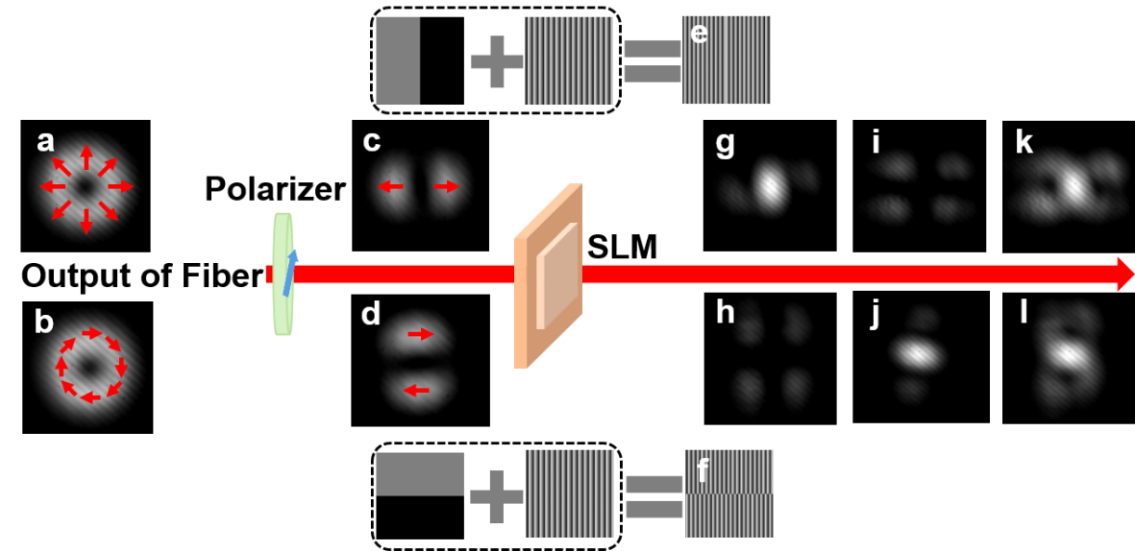


**Figure S9 |** Demultiplexing of TM_01_ and TE_01_ modes. Measured intensity distributions of (**a**) TM_01_ mode, (**b**) TE_01_ mode, (**c**) TM_01_ mode after a polarizer (horizontal polarizer axis), (**d**) TE_01_ mode after a polarizer, and (**g-l**) mode demultiplexing after an SLM. (**e**, **f**) Phase patterns loaded to the SLM for TM_01_ mode (**e**) and TE_01_ mode (**f**) demultiplexing. Top dashed box: superposition of a left-right 0/π pattern and a uniform grating pattern. Bottom dashed box: superposition of an up-down 0/π pattern and a uniform grating pattern. (**g**) Demultiplexing of TM_01_ mode using (**e**) when TE_01_ mode is absent (without crosstalk). (**h**) Demultiplexing of TM_01_ mode using (**f**) when TE_01_ mode is absent. (**i**) Demultiplexing of TE_01_ mode using (**e**) when TM_01_ mode is absent. (**j**) Demultiplexing of TE_01_ mode using (**f**) when TM_01_ mode is absent (without crosstalk). (**k**) TM_01_ demultiplexing from the multiplexed TM_01_ and TE_01_ modes using (**e**) (with crosstalk). (**l**) TE_01_ demultiplexing from the multiplexed TM_01_ and TE_01_ modes using (**f**) (with crosstalk).

10. Additional Results of Constellations of QPSK and 16-QAM Signals

For the data-carrying fiber vector eigenmodes (TM_01_, TE_01_) transmission through a 2-km LCF, shown in Fig. S10a and S10b are the measured constellations of 10-Gbit s^-1^ QPSK and 20-Gbit s^-1^ 16-QAM signals without crosstalk, i.e. only TM_01_ mode or TE_01_ mode is present. The constellations in the first and second rows correspond to QPSK and 16-QAM signals, respectively. The constellations in the left, middle and right columns correspond to back-to-back, TM_01_ mode from Chip1 after 2-km LCF transmission and TE_01_ mode from Chip2 after 2-km LCF transmission, respectively.


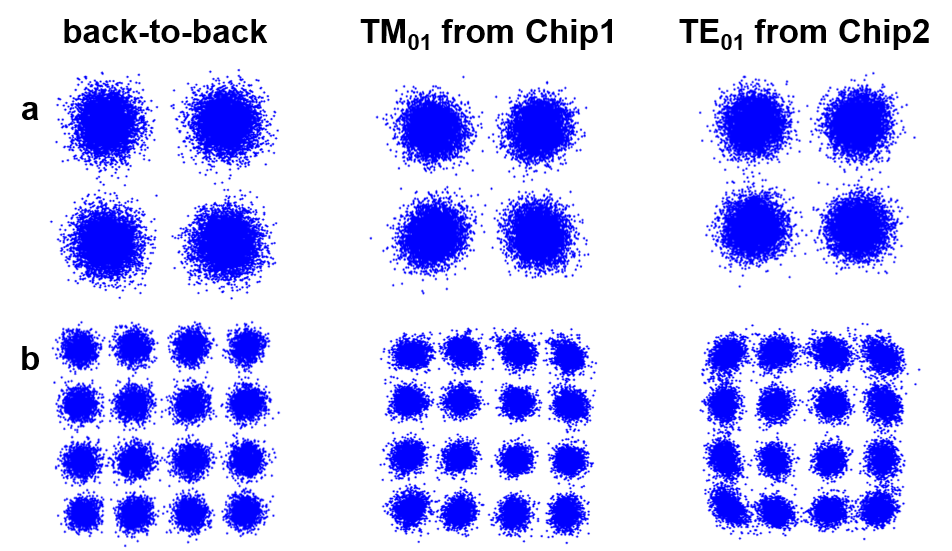


**Figure S10 |** Measured constellations of (**a**) 10-Gbit/s QPSK and (**b**) 20-Gbit/s 16-QAM signals without crosstalk. Left: back-to-back. Middle: TM_01_ from Chip1 after 2-km LCF transmission. Right: TE_01_ from Chip2 after 2-km LCF transmission.

11. Captions for Movies S1 to S4

**Movie S1** | Measured rotating intensity distribution of TM_01_ mode emitted from Chip1 after passing through a rotating polarizer in the direction indicated by the arrow.

**Movie S2** | Measured rotating intensity distribution of TE_01_ mode emitted from Chip2 after passing through a rotating polarizer in the direction indicated by the arrow.

**Movie S3** | Measured rotating intensity distribution of TM_01_ mode after propagating through a 2-km LCF followed by a rotating polarizer in the direction indicated by the arrow.

**Movie S4** | Measured rotating intensity distribution of TE_01_ mode after propagating through a 2-km LCF followed by a rotating polarizer in the direction indicated by the arrow.

**References**

1. Cherchi, M., Ylinen, S., Harjanne, M., Kapulainen, M. & Aalto, T. Dramatic size reduction of waveguide bends on a micron-scale silicon photonic platform. *Opt. Express* **21**, 17814–17823 (2013).
2. Driscoll, J. B. *et al.* Large longitudinal electric fields (Ez) in silicon nanowire waveguides. *Opt. Express* **17**, 2797–2804 (2009).
3. Cai, X. *et al.* Integrated compact optical vortex beam emitters. *Science* **338**, 363–366 (2012).
4. Moresco, M. *et al.* Method for Characterization of Si waveguide propagation loss. *Opt. Express* **21**, 5391–5400 (2013).
5. Gorodetsky, M. L. *et al.* Rayleigh scattering in high-Q microspheres. *J. Opt. Soc. Am. B* **17**, 1051–1057 (2000).
6. Zhang, Z., Dainese, M., Wosinski, L. & Qiu, M. Resonance-splitting and enhanced notch depth in SOI ring resonators with mutual mode coupling. *Opt. Express* **16**, 4621–4630 (2008).
7. Weiss, D. S. *et al.* Splitting of high-Q Mie modes induced by light backscattering in silica microspheres. *Opt. Lett* **20**, 1835–1837 (1995).
8. Haus, H. Waves and Fields in Optoelectronics (Prentice-Hall, Englewood Cliffs, N.J., 1984).
9. Zhu, J. *et al.* On-chip single nanoparticle detection and sizing by mode splitting in an ultrahigh-Q microresonator. *Nature Photon* **4**, 46–49 (2010).
